# *Babesia divergens* Shows Equal Predilection for Human ABO Blood Types in an In Vitro Erythrocyte Preference Assay

**DOI:** 10.3390/pathogens12060803

**Published:** 2023-06-05

**Authors:** Muyideen K. Tijani, Lena Danielsson, Jill R. Storry, Martin L. Olsson, Kristina E. M. Persson

**Affiliations:** 1 Cellular Parasitology Programme, Cell Biology and Genetics Unit, Department of Zoology, University of Ibadan, Ibadan 200132, Nigeria; muyideen_kolapo.tijani@med.lu.se; 2 Division of Clinical Chemistry and Pharmacology, Department of Laboratory Medicine, Lund University, 22185 Lund, Sweden; 3 Clinical Chemistry and Pharmacology, Laboratory Medicine, Office for Medical Services, Region Skåne, 22185 Lund, Sweden; 4 Division of Hematology and Transfusion Medicine, Department of Laboratory Medicine, Lund University, 22185 Lund, Sweden; 5 Department of Clinical Immunology and Transfusion Medicine, Office for Medical Services, Region Skåne, 22185 Lund, Sweden

**Keywords:** *Babesia divergens*, ABO, preference assay, blood type

## Abstract

*Babesia* is spread to humans via ticks or blood transfusions. Severity of *Plasmodium falciparum* malaria is strongly correlated to the ABO blood group of the patient. *Babesia divergens* is an intraerythrocytic parasite with many similarities to malaria, but the impact of ABO on the susceptibility to and progression of the infection in humans is unknown. We have now cultured *B. divergens* in human group A, B and O erythrocytes in vitro and measured rates of multiplication. The predilection for the different erythrocyte types was also determined using an in vitro erythrocyte preference assay when the parasites were grown in group A, B or O erythrocytes over time and then offered to invade differently stained erythrocytes of all the blood types at the same time. The results showed no difference in multiplication rates for the different blood types, and the parasite exhibited no obvious morphological differences in the different blood types. When cultured first in one blood type and then offered to grow in the others, the preference assay showed that there was no difference between the A, B or O blood groups. In conclusion, this indicates that individuals of the different ABO blood types are likely to be equally susceptible to *B. divergens* infections.

## 1. Introduction

Babesiosis is a zoonosis caused mainly by four *Babesia* species: *B. microti*, *B. duncani*, *B. venatorum* and *B. divergens* [1]. These parasites invade and replicate inside erythrocytes and some of the symptoms, such as fever and hemolytic anemia, overlap with those of malaria, which is caused by the related *Plasmodium* species. There are also morphological similarities between the parasites, which may sometimes cause misdiagnosis. *Babesia* can sometimes cause severe disease with multiorgan failure, especially in the elderly or in those who are asplenic or immunosuppressed. The parasites can spread through ticks or blood transfusion [1,2,3], and transfusion of immunologically weak individuals can lead to severe complications which can even be fatal [4].

Naturally, *Babesia* is transmitted through ticks of the Ixodidae family. *Ixodes ricinus* is mainly found in Europe, and three of the four known disease-causing *Babesia* species in humans have been demonstrated in *I. ricinus* [5]. Arthropod vectors seem to have evolutionary preferences for particular human ABO blood types. Therefore, it is convenient to assume that a vector’s preference may have a constraining effect on the blood type choices of the parasite it transmits. For instance, *Anopheles gambiae*, the main vector that transmits *P. falciparum* in sub-Saharan Africa, has been shown to prefer feeding on blood group O individuals [6]. Interestingly, a relatively recent study demonstrated that some *P. falciparum* laboratory strains have a higher propensity to infect group O than group A erythrocytes [7]. However, since fresh clinical isolates of *P. falciparum* often form rosettes, and isolates from patients with blood group O often show less rosetting, the total effect in humans of having blood group O might still be advantageous [8].

Emerging data suggest that mosquitoes’ blood type preferences may be influenced by genetics and/or geography, since *A. stephensi,* which is mainly distributed in Asia, has been shown to prefer blood group AB [9]. *I. ricinus* ticks collected from the wild in the Czech Republic demonstrated more attraction to group A blood [10]. Some studies have demonstrated that host antibodies reacting against vector antigens could affect feeding success and reproductive capacity of the vectors [11,12,13]. For instance, levels of naturally produced antibodies against a trisaccharide, Galα1-3Galβ1-4GlcNAc-R (α-gal), which is found in mosquito and tick saliva [14], is influenced by the ABO blood group since type B individuals produce less of these antibodies [15,16]. Such antibodies may complicate the ABO preference of mosquitoes or ticks, but the relationship between anti-α-gal antibodies and ABO blood group preference has not been demonstrated experimentally. A recent study, conducted in areas of the USA where *B. microti* is prevalent, found no association between ABO blood group in blood donors or patients and susceptibility to *B. microti* infection [17]. Currently, there is no information about the blood type preference of any of the *Babesia* species when studied on a cellular level grown in vitro, and no data on patients with *B. divergens*. This information is crucial to give us insights into the susceptibility to *B. divergens* infection for each individual patient, but also for the potential therapeutic advantages associated with different ABO blood types, if a patient needs a blood transfusion.

In this study, we determined the ABO blood group preference of *B. divergens* using an in vitro erythrocyte preference assay.

## 2. Materials and Methods

### 2.1. Babesia Divergens Culture

A *Babesia* isolate (Lund 1) from an infected bovine (cow) blood sample in the southern part of Sweden was introduced into in vitro routine cultures of 4% hematocrit of human erythrocytes in culture medium containing RPMI 1640-HEPES (Gibco, Life Technologies Ltd., Paisley, UK) with 1% AlbuMAX II, 25 μg/mL gentamicin, 5 mM L-glutamine and 200 μg/mL hypoxanthine (all from ThermoFisher Scientific, Auckland, NZ). Parasites were cultured at 37 °C in candle light boxes as described elsewhere [18,19]. Aliquots of the infected bovine blood sample were introduced directly into cultures containing human erythrocytes of blood groups A, B or O. Thin smears from the cultures were made at approximately 48 h and stained with Giemsa stain. Stained slides were viewed at 100× oil immersion objective magnification. The parasites underwent about 130 subcultures/passages over approximately nine months in the different blood groups before they were used in the assays. The isolate was confirmed to be *B. divergens* via PCR and sequencing at the Institute of Parasitology, University of Zurich, Switzerland.

### 2.2. Parasite Multiplication Rates in Different Blood Types

We used a starting parasitemia of 2–3% and 2% hematocrit of erythrocytes of the three ABO groups in separate wells containing 2 mL culture volume in 6-well culture plates, kept in humified candle light boxes at 37 °C, incubated for approximately 96 h. Parasitemia was determined every 48 h through staining 50 μL of the culture with 100 μg/mL acridine orange (Thermo Fisher Scientific, Rockford, IL, USA) for 30 min and then washing with PBS. Washed cells were fixed with 2% formaldehyde/0.2% glutaraldehyde in PBS for 1 h at 4 °C. After fixation, the cells were washed and resuspended in PBS and then analyzed via flow cytometry using an Accuri C6 flow cytometer (Becton Dickinson, BD Biosciences, NJ, USA). Acquired data were further analyzed using FlowJo (BD), version 10.8.1. Multiplication rates were expressed as a fraction of the starting parasitemia.

### 2.3. Erythrocyte Labelling and Preference Assay

Erythrocytes of the different blood groups were stained with different fluorescent dyes within 24–48 h of the collection from the donors. Small volumes of citrate-anticoagulated blood from leucocyte-filtered erythrocyte concentrates obtained following routine blood donation by 17 healthy anonymous donors, 6 of group A, 5 of group B and 6 of group O, obtained from the Department of Clinical Immunology and Transfusion Medicine, Office for Medical Services, Region Skåne, Lund, were used for this experiment. Erythrocytes of the three ABO groups were labelled through adding the cells (2% hematocrit) to 1 mL of RPMI 1640 containing either 1.5 μM Cell Trace far red, 5 μM Cell Trace CFSE or 5 μM Cell Trace yellow (all from Thermo Fisher Scientific, Rockford, IL, USA) followed by incubation at 37 °C, with intermittent mixing, for 1 h. Stained cells were washed with culture medium and resuspended in culture medium for further incubation at 37 °C for 30 min. Cells were then washed twice and finally resuspended in culture medium to 2% hematocrit. Fluorophores used to stain the erythrocytes were interchanged between the ABO blood groups so that no particular type was stained with the same fluorophore between consecutive experiments.

**Figure 1:**
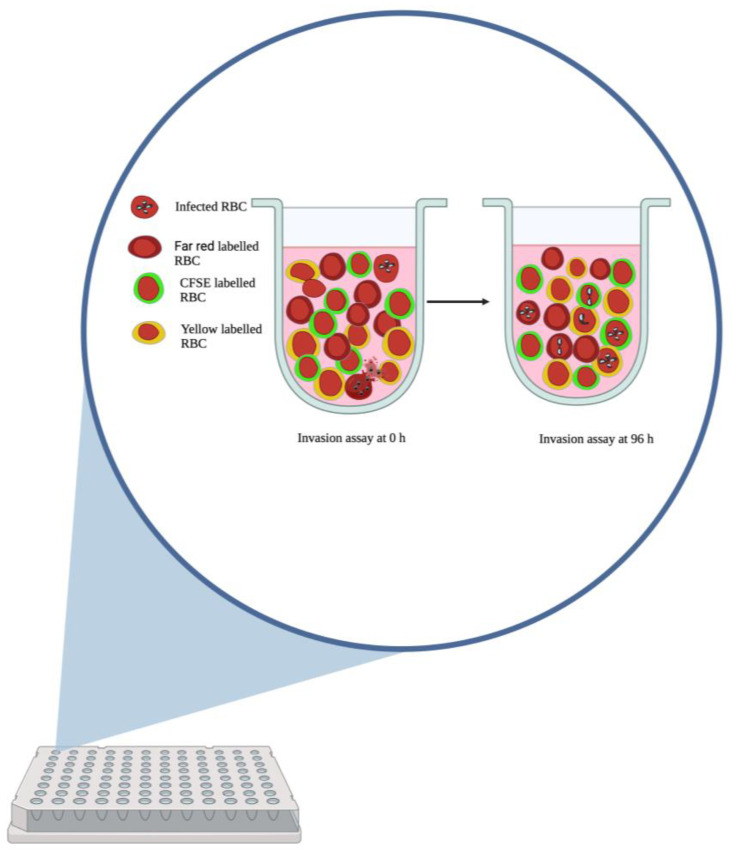
Diagrammatic representation of the use of differently labelled RBCs in an in vitro preference assay.

Equal volumes of labeled group A, B and O uninfected erythrocytes were mixed with *Babesia*-infected erythrocytes to obtain a 2% hematocrit and parasitemia of 1–2% (Figure 1). *B. divergens* parasites that had been adapted and cultured for about 9 months separately in erythrocytes of the three different ABO groups were used as parasite sources. Aliquots of 100 μL were placed in wells of round-bottom 96-well culture plates in duplicates and then incubated in a humidified candle light box for approximately 96 h. Fresh medium (10 μL) was gently mixed with the culture in each well at around 48 h of incubation. At the end of the assay, cells were washed with PBS and then stained with 2 μM Hoechst 33342 (Thermo Fisher Scientific, Rockford, IL, USA) in PBS for 1 h at 37 °C. Cells were fixed in 2% formaldehyde/0.2% glutaraldehyde in PBS for 1 h at 4 °C after washing with PBS. Cells were then washed and resuspended in PBS, followed by data acquisition using an LSR Fortessa X-20 (BD) flow cytometer. Further analyses were carried out using Flowjo software (BD). The percentage parasitemia obtained for each blood group was expressed as a fraction of the sum of percentage parasitemia for all the blood groups (A, B and O) and multiplied by 100. This normalization enabled comparison between experiments.

### 2.4. Statistical Analyses

Microsoft Excel version 16.72 (Microsoft Corporation, WA, USA) and GraphPad prism 9 (GraphPad Software, MA, USA) were used for data collation and statistical analyses, respectively. Median percentages of parasitemia between blood types were compared using the Kruskal–Wallis test. The log-rank test was used to compare trends of parasite multiplicity in different blood groups in a survival analysis.

## 3. Results

### 3.1. Invasion of Human Erythrocytes at 48 h

**Figure 2:**
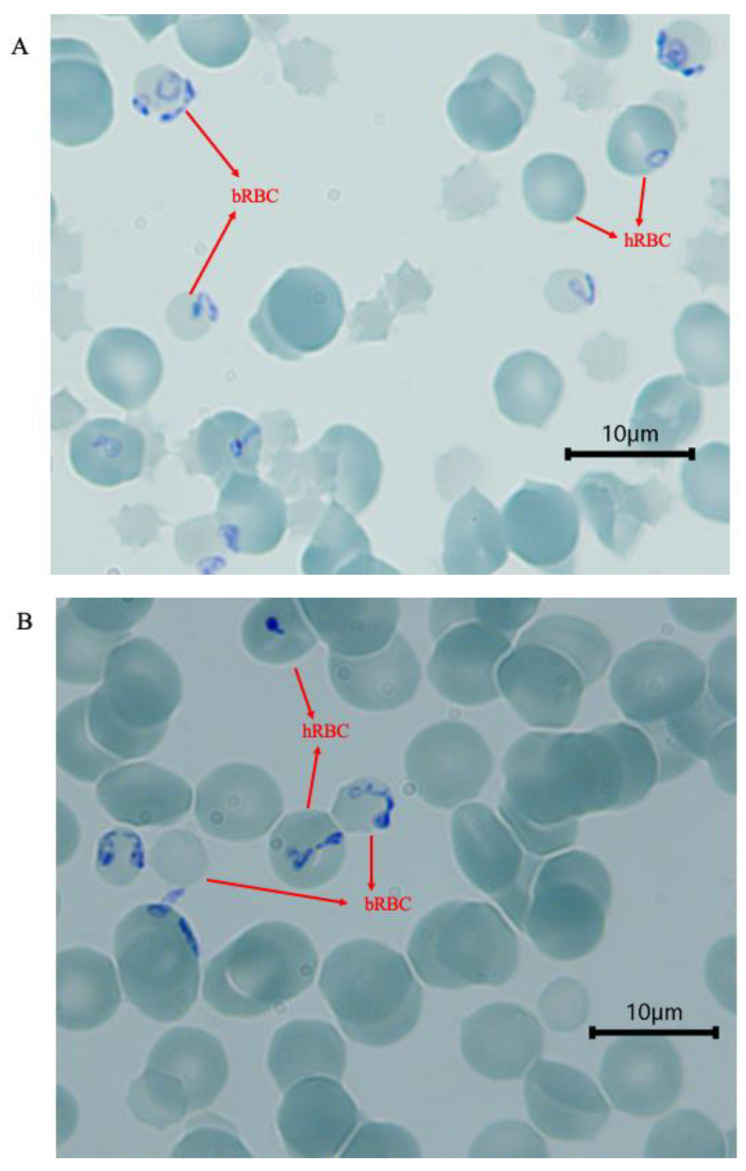

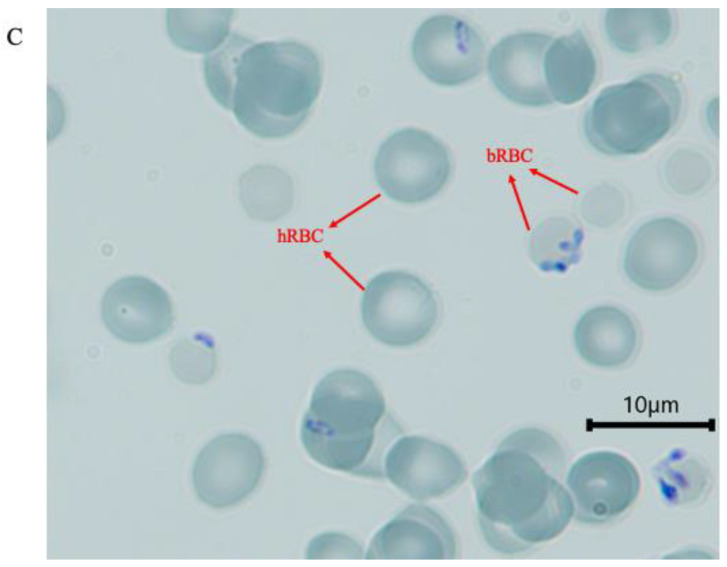
Images of Giemsa-stained thin films of *Babesia*-infected bovine erythrocytes mixed with human blood groups A (**A**), B (**B**) and O (**C**). Human erythrocytes have already been infected at 48 h after inoculation. hRBC and bRBC—human RBC and bovine RBC, respectively. The bovine erythrocytes in these images were smaller (about 4 μm in diameter) than human erythrocytes (about 7 μm in diameter).

When the parasites were inspected at 48 h, they had started to move from the infected bovine erythrocytes into human erythrocytes. There were no observable differences in the parasite morphology between blood groups A, B or O (Figure 2). Single and double trophozoites, paired pyriforms, double-paired pyriforms and tetrads were all seen at this time point (48 h). All observable stages including those formed by more than four parasites were eventually seen before the parasite isolate was used in assays described below.

### 3.2. Parasite Multiplication Rate in Erythrocytes of Different ABO Blood Groups

**Figure 3:**
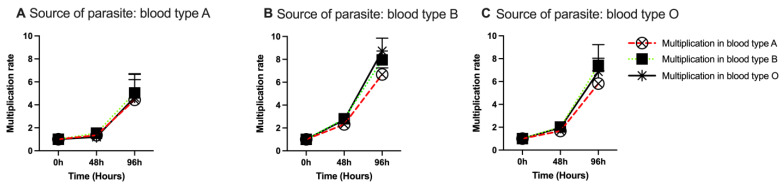
Multiplication of a *B. divergens* isolate in different blood types when the parasites that were used to start the culture had been grown only in erythrocytes of blood group A (**A**), B (**B**) or O (**C**). The cultures lasted for about 96 h and parasitemia was determined every 48 h. The bars indicate standard errors of the mean. The parasites multiplied steadily in the different ABO types, and there were no differences in the times for the parasites to double their starting parasitemia in the different blood types (*p* = 0.60).

The multiplication rate of *B. divergens* in erythrocytes of blood groups A, B and O was monitored over 4 days (96 h). The multiplication rate was similar in the different blood groups at 48 h, irrespective of which blood group the parasite that was used to start the culture had been adapted to. (Figure 3). The longitudinal sampling of the cultures provided the opportunity to compare the time taken to double the starting parasitemia (parasitemia on day 0) between the different blood groups. Survival analyses showed no difference in the ability of the parasite to double its population in the three blood types (*p* = 0.60).

### 3.3. Parasite ABO Blood Group Preference

**Figure 4:**
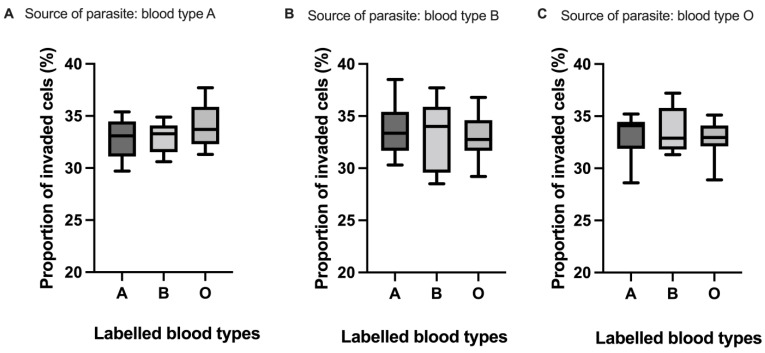
Comparison of median percentage proportions of labelled blood groups A, B and O invaded by a *B. divergens* isolate in a preference assay when the parasites that were used to start the assay had been grown only in blood group A (**A**), B (**B**) or O (**C**). The boxes represent the lower quartile, median and upper quartile values of percentage proportion of invaded cells. The whiskers represent the range. Comparison of median proportion of invaded cells via the Kruskal–Wallis test yielded no significant difference for all cases: A (*p* = 0.25), B (*p* = 0.88) and C (*p* = 0.78).

To gain more insight into main ABO blood group preferences of the *B. divergens* isolate used in this experiment, we further incubated the parasites with fluorescently labelled group A, B and O erythrocytes. This gives the parasite equal access to the labelled erythrocytes, and it could also help nullify the effects of differences in microenvironment when the different erythrocyte types were cultured with the parasites in separate wells. Additionally, to reduce any effects of adaptation to the blood group in which the parasites had been cultured in, we used parasites that had been cultured only in A, B, or O erythrocytes for the same duration of time as the parasite sources. *B. divergens* did not show preference for any of the blood types, as median percentage of invaded cells was not different between blood groups A, B or O (Figure 4A–C), whether the parasites that were used to start the assay had been cultured in A (*p* = 0.25), B (*p* = 0.88) or O (*p* = 0.78) blood types.

## 4. Discussion

Babesia is a parasite that is probably underdiagnosed in humans, and it is considered to be an emerging disease [20,21,22,23]. The ABO blood groups have been shown to affect human susceptibility to many diseases including infections [24,25,26]. Host–parasite interaction is often initiated through attachment of parasites to glycosylated cell surface receptors, so differential susceptibility to intracellular parasites or severity of the ensuing disease due to ABO antigens can easily be appreciated. There is lack of information about the blood group preferences of *B. divergens* that infect humans. We found no difference in the time taken by the *B. divergens* isolate studied here to double its population in the three main ABO blood groups, A, B and O. Already at 48 h after starting to invade human RBC, no differences could be seen in the microscope between the different blood groups. When multiplication rate experiments were performed after 9 months in each blood type, the parasites had for sure established themselves in the blood type they were growing in. Similar rates of multiplication as observed here may indicate that the ABO antigens do not affect the initial binding processes that lead to the invasion of erythrocytes by this *B. divergens* strain. Additionally, this parasite has potentially been growing in some rodent or deer before growing in the cattle, further making it plausible that it is a parasite that can adapt easily to different environments. Our results also suggest that any possible differences in the intrinsic nutrients between the ABO blood groups do not have any effects on the multiplication rate of the parasite. This study is in agreement with a recent clinical study on blood donors and patients admitted with *B. microti* infections, in which they could not find any association between ABO blood groups and risk of getting infected, having high peak parasitemias or intensive care unit admissions [17]. However, this same study found RhD+ individuals to be less susceptible to *Babesia* infection than RhD- individuals. Peak parasitemia was also significantly reduced among RhD+ individuals than RhD- individuals among babesiosis-hospitalized patients. Generally, immune status, age, sickle cell phenotype and asplenia are some of the other known factors that can affect the severity of babesiosis [2,4,27,28,29].

Furthermore, we used a method that allowed us to culture the *B*. *divergens* isolate with different blood types in the same well and to specifically determine parasitemia in the different blood types. Several *P. falciparum* laboratory strains have before been demonstrated to prefer blood group O+ using the same method [7]. The use of laboratory strains of *P. falciparum* that have traditionally been grown in group O blood may have influenced the choice of these parasites. In order to avoid this potential source of bias, we did not use laboratory strains of *Babesia* but a newly isolated *B. divergens*. Already after 48 h, we inspected the parasites in the microscope, but we could not detect any obvious differences when letting the parasites invade from bovine RBC to human RBC of blood groups A, B or O. We then let the parasites grow for 9 months in the different blood types and could show clearly for the first time that a *B. divergens* parasites did not show a clear preference for any of the ABO blood groups, irrespective of the blood group of the erythrocytes which the parasite had first been adapted to. This shows an extensive adaptability of *B. divergens* in invading erythrocytes through probably using different surface receptors. This might not be surprising considering the broad host range of *I. ricinus* that transmits *B. divergens*. The vector can feed on birds, hares, cattle, small rodents, deer and humans [30,31,32,33]. A preference for blood group A or O could perhaps be expected considering the preponderance of these blood types in European populations [34] where *B. divergens* is mainly transmitted, but *B. divergens* is also a natural parasite of cattle, a group of animals with elaborate but different surface glycans on bovine erythrocytes. Meanwhile, *I. ricinus* has been shown to prefer human blood group A in an in vitro experiment [10], but unlike mosquitoes that actively seek for preferred human blood types to feed on and thus can influence the parasites they carry, *Ixodidae* ticks are comparatively passive in their search for blood meals as they simply wait on vegetation to attach to any passing host. Therefore, the ability of *B. divergens* parasites to invade bovine and human erythrocytes of any type with relative ease might be its major evolutionary success as a parasite. Indeed, *B. divergens* has been shown to be able to establish itself with relative ease in vitro in bovine, human, ovine and equine erythrocytes [35].

*B. divergens* is usually cultured in group A erythrocytes in most laboratories [36,37,38]. Our results show that this parasite can also just as easily be cultured in group O and B erythrocytes. Culturing parasites in group O might be better if plasma is going to be added to cultures, for example, in growth assays, since some individuals might carry anti-A or anti-B antibodies that could cause agglutination. This work has provided insights into the nature of the interaction of *B. divergens* and the human ABO blood group system, and it also suggests that the risk of getting a severe disease is probably similar, whatever blood group the patient has.

Our conclusion is that ABO antigens are not protective when investigating *B. divergens*. Individuals of different blood groups are probably equally susceptible to both natural infection via ticks or infection through blood transfusion. More studies involving *Babesia* parasites of variable species, strains and genetic or geographic background are required to generate more useful information in this area. Whilst we investigated here for the first time the influence of ABO on *Babesia* invasion and multiplication parameters, there are numerous other blood group systems that are independently inherited but not routinely typed. Some of them have been implicated in other host–pathogen interactions, either as receptors or other susceptibility or resistance factors [17,39,40] and may constitute future targets of study. Documentation of the blood groups of babesiosis patients and any associated unique or severe reactions across different regions will also help to enhance our knowledge about the interactions of this important parasite with humans.

## Data Availability

All data is available in this paper, for exact numbers data is available upon reasonable request to the corresponding author.
